# The mitochondrial Cu^+^ transporter PiC2 (SLC25A3) is a target of MTF1 and contributes to the development of skeletal muscle *in vitro*

**DOI:** 10.3389/fmolb.2022.1037941

**Published:** 2022-11-09

**Authors:** Cat McCann, Michael Quinteros, Ifeoluwa Adelugba, Marcos N. Morgada, Aida R. Castelblanco, Emily J. Davis, Antonio Lanzirotti, Sarah J. Hainer, Alejandro J. Vila, Juan G. Navea, Teresita Padilla-Benavides

**Affiliations:** ^1^ Department of Molecular Biology and Biochemistry, Wesleyan University, Middletown, CT, United States; ^2^ University of Massachusetts Chan Medical School, Worcester, MA, United States; ^3^ Instituto de Biología Molecular y Celular de Rosario, Rosario, Argentina; ^4^ Department of Chemistry, Skidmore College, Saratoga Springs, NY, United States; ^5^ Center for Advanced Radiation Sources, The University of Chicago, Chicago, IL, United States; ^6^ Department of Biological Sciences. University of Pittsburgh, Pittsburgh, PA, United States

**Keywords:** SLC25A3, PiC2, MTF1, copper transport, cytochrome c oxidase, mitochondria

## Abstract

The loading of copper (Cu) into cytochrome c oxidase (COX) in mitochondria is essential for energy production in cells. Extensive studies have been performed to characterize mitochondrial cuproenzymes that contribute to the metallation of COX, such as Sco1, Sco2, and Cox17. However, limited information is available on the upstream mechanism of Cu transport and delivery to mitochondria, especially through Cu-impermeable membranes, in mammalian cells. The mitochondrial phosphate transporter SLC25A3, also known as PiC2, binds Cu^+^ and transports the ion through these membranes in eukaryotic cells, ultimately aiding in the metallation of COX. We used the well-established differentiation model of primary myoblasts derived from mouse satellite cells, wherein Cu availability is necessary for growth and maturation, and showed that PiC2 is a target of MTF1, and its expression is both induced during myogenesis and favored by Cu supplementation. PiC2 deletion using CRISPR/Cas9 showed that the transporter is required for proliferation and differentiation of primary myoblasts, as both processes are delayed upon PiC2 knock-out. The effects of PiC2 deletion were rescued by the addition of Cu to the growth medium, implying the deleterious effects of PiC2 knockout in myoblasts may be in part due to a failure to deliver sufficient Cu to the mitochondria, which can be compensated by other mitochondrial cuproproteins. Co-localization and co-immunoprecipitation of PiC2 and COX also suggest that PiC2 may participate upstream in the copper delivery chain into COX, as verified by *in vitro* Cu^+^-transfer experiments. These data indicate an important role for PiC2 in both the delivery of Cu to the mitochondria and COX, favoring the differentiation of primary myoblasts.

## 1 Introduction

Copper (Cu) is an essential redox cofactor for enzymatic and energy production reactions (Linder and Hazegh-Azam, 1996; Fraústo da Silva and Williams, 2001). Cu can also be toxic at high concentrations and, if deleteriously oxidized, enhances the production of reactive oxygen species (Prousek, 2007). Consequently, cells *must* control Cu levels, which they do *via* a complex cellular network of transmembrane transporters, soluble chaperones, and transcription factors (Lutsenko et al., 2007; Robinson and Winge, 2010; Festa and Thiele, 2011; Argüello et al., 2013; Ohrvik and Thiele, 2014). These proteins have high binding affinities for Cu, and they simultaneously chelate free Cu in the cells and distribute it to organelles and acceptor proteins (O'Halloran and Culotta, 2000; Rae et al., 1999). To complete this process, Cu is transferred through direct protein-protein interactions or mechanisms involving ligand exchange (O'Halloran and Culotta, 2000; Waldron et al., 2009; Barry et al., 2011; Otoikhian et al., 2012). Cu-transportin proteins enable Cu entry into and export out of the cell (Otoikhian et al., 2012; Padilla-Benavides et al., 2013a; Padilla-Benavides et al., 2014). Copper transporter 1 (CTR1) facilitates Cu entry to the cell (Lee et al., 2002; Ohrvik and Thiele, 2014). As Cu enters the cell, it is bound by acceptors, such as glutathione, antioxidant protein 1 (ATOX1), and the Cu-chaperone for superoxide dismutase (CCS) (Maryon et al., 2013). Some examples of cuproenzymes in eukaryotes are cytochrome *c* oxidase (COX), Cu, Zn-Superoxide dismutase (SOD1), tyrosinase, lysyl oxidase, and peptidyl-glycine-α-monooxygenase. These proteins may be cytosolic, mitochondrial, or secreted *via* the *trans*-Golgi network. The ATP-driven Cu-transporters ATP7A and ATP7B are responsible for the metallation of the secreted cuproproteins, as they transport cytosolic Cu into the lumen of the trans-Golgi network (Robinson and Winge, 2010; Festa and Thiele, 2011; O'Halloran and Culotta, 2000; Lutsenko, 2016). Failure in Cu acquisition, distribution, or delivery to appropriate acceptors leads to fatal disorders, such as Menkes’ and Wilson’s diseases (Daniel et al., 2004; Lutsenko et al., 2007; Graper et al., 2014). Moreover, cellular respiration depends on the fine-tuning of redox processes, which is finalized by the reduction of O_2_ mediated by COX, where Cu is an essential component for electron transfer.

The skeletal muscle is a contractile tissue composed of multinucleated myofibers formed by the fusion of differentiating muscle cells. Myoblast proliferation is largely regulated by the transcription factor Pax7 (Yin et al., 2013; Brack and Rando, 2012; Montarras et al., 2013; Buckingham and Rigby, 2014; Chang and Rudnicki, 2014; Padilla-Benavides et al., 2015; Motohashi and Asakura, 2014; Sambasivan and Tajbakhsh, 2015; Seale et al., 2004; Seale et al., 2000; von Maltzahn et al., 2013), and myogenic differentiation is dependent on *Pax7* downregulation, induction of the master regulator *myogenin*, and myoblast fusion to create myofibers (Zammit et al., 2004). Muscle tissue has an intrinsically high number of mitochondria (Moyes et al., 1997; Remels et al., 2010) and, consequently, a high demand for Cu. As such, skeletal muscle differentiation serves as a good physiological model in which to study mitochondrial Cu transport and the role of Cu in mitochondrial biogenesis. Myogenesis results in metabolic and morphological changes that have a strong link to Cu biology.

It is well-established that Cu is required for the maturation and activity of COX (Leary et al., 2004; Morgada et al., 2015). However, there are still gaps in our understanding of the pathway(s) responsible for the delivery of Cu across the mitochondrial membranes, and the mechanisms by which it is subsequently loaded into COX. In *Saccharomyces cerevisiae*, a non-proteinaceous trafficking system supports mitochondrial Cu transport: a cytosolic Cu ligand (CuL) delivers the ion to the organelle. Therein, the yeast mitochondrial phosphate carrier yPiC2 imports Cu into the matrix, which is eventually utilized by COX and SOD1 (Vest et al., 2013). Limited information is available on mitochondrial Cu-transporting systems for other eukaryotes. Sequence homology analyses showed that the human and murine mitochondrial phosphate transporter encoded by *SLC25A3* has 48% identity to yPiC2. SLC25A3, also called PiC2, is highly conserved in mammals, and it catalyzes the transport of P_i_ into the mitochondrial matrix (Seifert et al., 2015). Early homology models suggested that SLC25A3 has six transmembrane (TM) segments, 3-fold symmetry, and N- and C-termini facing the intermembrane space (Runswick et al., 1987). SLC25A3 has two isoforms that arise from alternative splicing of exon 3: PiC2A, which is expressed exclusively in cardiac and skeletal muscle, and PiC2B, which is ubiquitously expressed (Dolce et al., 1994; Dolce et al., 1996). Biochemical analysis of the bovine PiC2 (bPiC2) transporter showed that the bPiC2A isoform has higher P_i_ transport affinity than bPiC2B, whereas bPiC2B has a higher maximal transport rate (Fiermonte et al., 1998). Experimental evidence strongly suggests that the principal activity of PiC2 is to supply P_i_ for oxidative phosphorylation, and mutations in this transporter lead to a failure in phosphate transport that impacts muscle function and/or development. Mammalian PiC2 has also been proposed as a mitochondrial Cu transporter, importing Cu through the outer mitochondrial membrane and into the matrix (Boulet et al., 2018). Knockdown of PiC2 or depletion of Cu in the mitochondrial matrix results in defects in COX activity. Moreover, mutations in PiC2A have been linked to several diseases, including lactic acidosis, hypertrophic cardiomyopathy, and muscular hypotonia, due to a muscle-specific deficiency in ATP synthesis (Mayr et al., 2007; Mayr et al., 2011; Boulet et al., 2018).

Given these data, we hypothesized that PiC2 plays a role in Cu delivery to the mitochondria, and subsequently COX, that is important for muscle development and function. We used cultured primary myoblasts derived from mouse satellite cells to investigate the role that the murine PiC2 (mPiC2) plays in mitochondrial Cu delivery and skeletal muscle growth and differentiation. We found that mPiC2 expression is induced during muscle differentiation, and that this induction is significantly increased in the presence of Cu. Bioinformatic analyses of publicly available ChIP-seq datasets (Tavera-Montanez et al., 2019) for differentiating myoblasts showed that *PiC2* is a target of the metal-regulatory transcription factor (MTF1). MTF1 primarily regulates the expression of metal binding and transporting proteins to maintain homeostasis of these metals, including Cu (Tavera-Montanez et al., 2019; Maung et al., 2021). We previously found that MTF1 also targets genes involved in skeletal muscle differentiation, such as *myogenin*, and that knockdown of MTF1 results in impairments in the differentiation of myoblasts (Tavera-Montanez et al., 2019). Moreover, this regulation is also enhanced by the supplementation of Cu (Tavera-Montanez et al., 2019). Homology modeling using AlphaFold showed that mPiC2 has several potential metal binding residues in the transmembrane domain. Stoichiometric determination of recombinant hPiC2 revealed that PiC2A seems to bind two equivalents of Cu, while PiC2B seems to bind three equivalents of Cu per transporter. In addition, immunohistochemical and immunoprecipitation analyses showed that mPiC2 interacts with several mitochondrial cuproproteins, and *in vitro* Cu^+^ transfer experiments showed that PiC2A can deliver the ion to COX directly. Finally, CRISPR/Cas9 mediated deletion of *mPiC2* delayed myoblast growth and differentiation, which may be partially explained by the decreased expression of various mitochondrial cuproproteins in these cells. These proliferation and differentiation defects, and deficiency in cuproprotein expression was recovered by supplementation of copper to the culture media. Together, these data strongly suggest that mPiC2 is a novel component of a mitochondrial machinery that may contribute to the metallation of COX, and is required for the maturation of the skeletal muscle lineage.

## 2 Materials and methods

Primary myoblasts culture – Satellite cells were isolated from the leg muscle of 3–6 week-old wild type C57Bl/6 mice as previously described (Tavera-Montanez et al., 2019). The cells were isolated as part of a previous research protocol which was reviewed and approved by the animal care facility at the University of Massachusetts Chan Medical School (Worcester, MA, United States) in accordance with the Institutional Animal Care and Use Committee guidelines. The corresponding author was based at this institution when the original isolation was conducted. The tissue was extracted, fragmented, washed with HBSS (Thermo Fisher Scientific, Waltham, MA, United States) and treated with 0.1% Pronase for 1 h at 37°C. The cells were filtered using a 100-µm sieve and resuspended in 3 ml of growth medium containing 1:1 v/v Dulbecco’s modified Eagle’s medium (DMEM):F-12, 20% fetal bovine serum (FBS), 5% chicken embryo extract, 25 ng/ml of basic fibroblast growth factor (FGF) and 1% antibiotics. The cells were filtered again using a 40-µm cell sieve and centrifuged at 1,000 x *g* for 1 min at room temperature (RT). The cells were separated by Percoll step-gradient (35% and 70%) and centrifuged for 20 min at 1,850 x *g* at RT. The myoblasts located at the lower interface of the 70% Percoll fraction were washed with HBSS, centrifuged for 5 min at 1,000 x *g*, and resuspended in growth medium for plating.

Samples for proliferating myoblasts were isolated and processed for further experimentation after 48 h of seeding in growth media. To induce differentiation, the myoblasts were seeded on plates coated with 0.02% collagen (Advanced BioMatrix, Carlsbad, CA, United States) (Nasipak et al., 2015). The differentiation treatments are indicated in the figures and were performed as previously described (Vest et al., 2018; Tavera-Montanez et al., 2019). Briefly, the differentiation medium was supplemented or not with insulin, and then with CuSO_4_ or the chelator tetraethylenepentamine (TEPA), both at concentrations of 100 µM for proliferating myoblasts and 30 µM for differentiating myoblasts.

HEK293T cells were purchased from ATCC (Manassas, VA) and were maintained in growth media containing DMEM supplemented with 10% FBS and 1% penicillin-streptomycin in a humidified incubator at 37^°^C with 5% CO_2_.

Plasmid construction, virus production, and transduction of primary myoblasts -CRISPR/Cas9 plasmid construction was performed using three custom-designed sgRNAs to recognize the intron/exon junction 2 of the *Slc25a3* (*PiC2*) murine gene (Reference Sequence: ENSMUSG00000061904). Each gRNA consisted of 20 nucleotides complementary to the sequence that precedes a 5′-NGG protospacer-adjacent motif (PAM) located in the targeted intron. Specificity was validated by a search through the entire genome to avoid off-target effects. The sequences of the gRNAs designed were: PiC2-gRNA2_Intr_F 5′- CAC​CGC​AAC​AAT​ACA​AAC​CTG​CAT​G -3′ and PiC2-gRNA2_Intr_R 5′- AAA​CCA​TGC​AGG​TTT​GTA​TTG​TTG​C -3’. Preparation of CRISPR/Cas9 lentiviral constructs was performed using the lentiCRISPRv2 oligo cloning protocol (Sanjana et al., 2014). Briefly, sense and antisense oligos obtained from Integrated DNA Technology (IDT), were annealed and phosphorylated to form double stranded oligos. They were then cloned into the BsmBI–BsmBI sites downstream from the human U6 promoter of the lentiCRISPRv2 plasmid kind gift from Dr. F. Zhang; Addgene plasmid # 52961) (Sanjana et al., 2014; Shalem et al., 2014). The empty plasmid that expresses only Cas9 but no sgRNA was included as a null knockout control.

To generate Lentiviral particles, 5 × 10^6^ HEK293T cells were plated in 10 cm dishes. The next day, transfection was performed using 15 µg of the sgRNA-containing CRISPR/Cas9 constructs mixed with the packing vectors pLP1 (15 µg), pLP2 (6 µg), pSVGV (3 µg). Transfections were performed using Lipofectamine 2000 according to the manufacturer’s instructions (Invitrogen). The media was changed the next day to 10 ml DMEM (Life Technologies) supplemented with 10% FBS (Life Technologies). The viral supernatant was harvested after 24 h and 48 h of incubation and filtered through a 0.22 µm syringe filter (Millipore). To infect primary myoblasts, 5 ml of the filtered supernatant supplemented with 8 μg/ml polybrene (Sigma) were used to transduce 2 × 10^6^ cells. After overnight incubation, infected cells were then selected in growth media containing 2 μg/ml puromycin (Invitrogen). Stable myoblasts were maintained in growth media containing 1 μg/ml puromycin.

Parallel 1 plasmids encoding the human *PiC2* isoforms A and B labeled with an N-terminus hexa-histidine tag for protein expression were a generous gift from Dr. Paul Cobine (Department of Biological Sciences, Auburn University) and have been previously described (Sheffield et al., 1999; Boulet et al., 2018).

Antibodies - The primary antibodies used from Abclonal Technologies (Woburn, MA) were rabbit anti-GAPDH (A19056), anti-PiC2 (custom made against the 38–47 residues “GQPRRPRNLA” from the human protein, which cross reacts with the murine protein), anti-SCO1 (A6734), anti-SCO2 (A7051), anti-COX1 (MT-CO1, A17889, for co-IP experiments), anti-ATP5A1 (ATP synthase, A5884) and the mouse anti-His-Tag (AE003). The mouse anti-MTF1 (H-6, sc-365090), anti-sarco/endoplasmic reticulum Ca^2+^-ATPase (SERCA, sc-271669), anti-COX2 (D-5, sc-514489) primary antibodies were from Santa Cruz Biotechnologies (Dallas, TX). The mouse anti-complex I primary antibody (18G12BC2) was from ThermoFisher Scientific. Hybridoma supernatants against Pax7, anti-myosin heavy chain (MF20), and Myogenin (F5D) were obtained from the Developmental Studies Hybridoma Bank (University of Iowa; deposited by A. Kawakami, D. A. Fischman and W. E. Wright, respectively).

The secondary HRP-conjugated anti-mouse and anti-rabbit antibodies were from Invitrogen (31,430 and 31,460, respectively) and the fluorescent goat anti-rabbit Alexa-488 and anti-mouse Alexa-594 secondary antibodies were from ThermoFisher (A-11008 and A-21203, respectively).

Primary myoblast immunofluorescence - Primary myoblasts used in immunofluorescence experiments were grown on glass bottom Cellview Advanced TC culture dishes (Greiner Bio One). Samples were obtained for proliferation and at 24 h, 48 h, and 72 h after induction of differentiation. Cells were fixed in 10% formalin, permeabilized with PBT buffer [0.5% Triton-X100 in phosphate buffered saline (PBS)] and blocked in 5% horse serum prepared in PBT. Cells were incubated with the indicated primary antibodies (1:100) in blocking solution overnight at 4°C. The samples were then washed three times with PBT solution for 10 min at RT. Then, the cells were incubated with the goat anti-rabbit Alexa-488 secondary antibody (1:500) in blocking solution for 2 h at RT and 30 min with DAPI. Images were taken with a Leica TCS SP5 Confocal Laser Scanning Microscope (Leica) using a 40X water immersion objective. Fluorescence intensity of the images depicted was measured using Fiji (National Institute of Health) (Schindelin et al., 2012) and normalized to proliferating cells values. The Pearson correlation coefficient was used to measure the strength of a linear association between Pic2 and COX1 in confocal images, it is denoted by *r* and was calculated with Fiji.

Gene expression analyses - Three independent biological replicates of proliferating and differentiating primary myoblasts were washed with ice cold PBS prior to RNA extraction using Trizol (Invitrogen). cDNA synthesis was performed using 1 μg of RNA, DNase I amplification grade (Invitrogen 18,068–015), and Superscript III (Invitrogen 18,080–400) according to the manufacturer’s instructions. Changes in gene expression were analyzed by quantitative RT-PCR using Fast SYBR-Green master mix (ThermoFisher Scientific) on the ABI StepOne Plus Sequence Detection System (Applied Biosystems) using the comparative Ct method (Livak and Schmittgen, 2001) and *Ef1*α as a control. The primers used for *mPiC2*: forward 5′- CTG​GTG​CAC​GAT​GGC​CTG-3′ and reverse 5′-AAC​CAA​GCT​GTA​TGT​GT-3’; *Ef1α:* forward 5′-AGC​TTC​TCT​GAC​TAC​CCT​CCA​CTT-3′ and reverse 5′-GAC​CGT​TCT​TCC​ACC​ACT​GAT​T-3′.

Western blot analyses - Proliferating and differentiating primary myoblasts were washed with PBS and solubilized with RIPA buffer (10 mM PIPES, pH 7.4,150 mM NaCl, 2 mM EDTA, 1% Triton X-100, 0.5% sodium deoxycholate, and 10% glycerol) containing Complete Protease Inhibitor. Lysates were then sonicated ten times on high power for 30 s and left to rest for 30 s. Protein concentrations were determined using Bradford assay (Bradford, 1976). Twenty micrograms of protein were separated on 10% SDS gels and then transferred to polyvinylidene difluoride (PVDF; Millipore) membranes. Membranes were blocked for 1 h at RT in blocking buffer (Biorad) and then incubated overnight in the indicated primary antibodies diluted 1:1,000 in PBS. Membranes were washed three times in PBST and then incubated for 2 h at RT in the indicated secondary antibodies diluted 1:1,000 in PBS. Following two washes in PBST and one wash in PBS, membranes were treated with horseradish peroxidase (HRP) substrate for enhanced chemiluminescence (ECL; Tanon), and then imaged on an Analytik Jena imager. GAPDH was used as a loading control.

Chromatin immunoprecipitation analyses - Three independent biological replicates of proliferating and differentiating primary myoblasts were crosslinked with 1% formaldehyde and incubated for 10 min at RT with continuous mild shaking. Glycine was used to inactivate the fixative reagent and cells were incubated for 5 min at RT. Samples were washed 3 times with 10 ml of ice-cold PBS supplemented with Complete Protease Inhibitor (Roche, Basel, Switzerland) and cells were resuspended in 1 ml of ice-cold PBS supplemented with Complete Protease Inhibitor. Samples were centrifuged for 5 min at 5,000 × *g* at 4°C and the PBS discarded. Primary myoblasts were lysed using the SimpleChIP Plus Sonication Chromatin IP Kit (Cell Signaling Technology), following the manufacturer’s instructions. Proliferating cells were sonicated using a Bioruptor UCD-200 (Diagenode, Denville, NJ, United States) for three cycles of, 30 s on and 30 s off at mild intensity and 5 cycles for differentiating myoblasts. The samples were then incubated with the anti-MTF1 antibody or IgG as a negative control (Tavera-Montanez et al., 2019). Immunoprecipitated material was collected with magnetic beads, and washed with 1x ChIP buffer supplemented with NaCl, as recommended by the manufacturer. Samples were eluted in 1x elution buffer, incubated overnight at 65°C and reverse crosslinked with NaCl. The resulting DNA was purified using the ChIP DNA clean concentrator, following the manufacturer’s instructions (Zymo Research, Irvine, CA, United States). The DNA was stored at −80°C until further analysis by semiquantitative real-time PCR (qPCR). The primer sequences in the promoter region of *mPiC2* were: forward 5′-GGA​CGT​CAT​CGC​GTC​CTC-3′ and reverse: 5′-AGG​TGA​CCT​ACT​CAC​TCT-3′.

The ChIP-seq data analyzed here for PiC2 was previously made publicly available with the GEO accession number: GSE116331, and can be downloaded here: https://www.ncbi.nlm.nih.gov/geo/query/acc.cgi?acc=GSE116331.

Sequence analyses and modeling of the putative metal binding sites of PiC2 (SLC25A3) – Protein sequence analyses were performed using the murine and human sequences for PiC2. These were aligned with MUSCLE (Edgar, 2004) and ESPript software (Gouet et al., 1999; Robert and Gouet, 2014). Due to the absence of a crystal structure for the transporter, we used Alpha Fold structure prediction software (Jumper et al., 2021; Varadi et al., 2022) to obtain a model of the human PiC2 protein (Uniprot accession number: A0A024RBE8). The conserved putative copper binding residues in this model are highlighted in green.

Cloning, expression, and purification of proteins - Human PiC2A and PiC2B were previously cloned (Boulet et al., 2018) into the pHis-Parallel1 vector which adds a N-terminal 6His-tag (Sheffield et al., 1999) and then transformed into *Escherichia coli* BL (Mauro, 1961), (DE3) competent cells. Protein expression was performed followed the autoinducing media protocol (Studier, 2005). Purification of PiC2 proteins followed an established membrane protein purification procedure using a Ni-NTA column to elute the His-tagged proteins (Mandal and Argüello, 2003; Gonzalez-Guerrero and Arguello, 2008; Padilla-Benavides et al., 2013a; Padilla-Benavides et al., 2013b; Blaby-Haas et al., 2014; Raimunda et al., 2014). Cells were suspended in buffer A (25 mM Tris, pH 7.0, 100 mM sucrose, 1 mM phenylmethylsulfonyl fluoride (PMSF; Sigma) and disrupted with a French press at 20,000 p. s.i. Cell debris was removed by centrifugation at 8,000 × *g* for 20 min at 4^°^C. To pellet the membranes, the supernatant was then centrifuged at 229,000 × *g* for 1 h at 4^°^C, and then resuspended in buffer A at a concentration of 10 mg/ml–15 mg/ml. Membrane proteins were diluted and solubilized to a final concentration of 3 mg/ml in buffer B (25 mM Tris, pH 8.0, 100 mM sucrose, 500 mM NaCl, 1 mM PMSF) containing 0.75% dodecyl-β-D-maltoside (DDM; Calbiochem). Then incubated for 1 h at 4°C with mild agitation and centrifuged at 229,000 × *g* for 1 h. The supernatant was incubated at 4°C overnight with Ni^2+^-nitrilotriacetic acid resin (Qiagen) pre-equilibrated with buffer B containing 0.05% DDM, and 5 mM imidazole. The next day, the samples were washed with buffer B containing 0.05% DDM and 20 mM imidazole, and the protein was eluted with buffer B, 0.05% DDM, 250 mM imidazole. Fractions were concentrated and buffer exchange was performed using 10 kDa cut-off centricons (Millipore) to final concentrations of 25 mM Tris pH 8.0, 100 mM sucrose, 50 mM NaCl, and 0.01% DDM (buffer C).

A stable chimera encoding cytochrome *c* oxidase (apoCuA) was expressed and purified by chromatography as previously described (Morgada et al., 2015; Leguto et al., 2019). Briefly, a 10 ml culture of BL21 (DE3)-pET-9a/CuATt3L was grown overnight in LB medium, supplemented with 50 μg/ml of kanamycin at 37°C. This bacterial culture was used to inoculate 5 L–6 L cultures and allowed to grow at 37°C for 2 h–2.5 h until reaching an OD_600_ of 0.8. Protein expression was induced by adding 1 mM Isopropyl b- d -thiogalactopyranoside (IPTG) and incubated at 37°C for 5 h–6 h or 30°C overnight. Bacteria were then pelleted at 5,000 rpm for 15 min and resuspended in 30 ml of Tris-HCl 50 mM, pH 8.0 supplemented with 2 mM PMSF, 40 µl of DNAse (stock 10 mg/ml), and 5 mM MgCl_2_. Cells were then lysed by sonication (5 pulses of 30 s each, with 1 min pauses) at maximum power. Samples were heated at 55°C–60°C for 10 min–15 min until a precipitate appeared, and then centrifuged at 20,000 rpm, at 4°C. Supernatants were collected and DNA was further precipitated by adding cold streptomycin sulfate. Samples were centrifuged again at 20,000 rpm, at 4°C and the supernatant was collected for further fractionation with ammonium sulfate. After another centrifugation step at 13,000 rpm for 5 min at 4°C the pellet containing the protein was resuspended in 5 ml 50 mM Tris pH 8.0. A final precipitation of remaining cell debris was performed by adding 1 ml of ice cold 5 M sodium acetate pH 8.0 every 10 ml of extract and centrifugation at 13,000 rpm for 10 min at 4°C. A neutralization step follows by using 100 µl of 5 M NaOH was added for every 1 ml of sodium acetate added. Samples were then dialyzed to eliminate salts by adding 5 mM DTT and 5 mM EDTA against 50 mM Kpi pH 7.5. The next day, the proteins were collected from the dialysis bag and further purified by chromatography on a Q-sepharose Fast Flow column in 50 mM Kpi pH 7.5 containing 1 mM DTT by collecting the CuA protein in the flowthrough. Proteins were aliquoted and stored in buffer containing 100 mM Pi pH 6.0, 100 mM KCl, and 1 mM DTT at −20ºC until further use. All protein concentrations were determined using Bradford assay (Bradford, 1976).

Cu^+^ loading to PiC2 and metal binding analyses - Cu^+^ loading was performed by incubating 20 µM of each apo-protein in the presence of a 10 M excess of CuSO_4_, 25 mM HEPES pH 8.0, 150 mM NaCl, and 10 mM ascorbate for 10 min at RT with gentle agitation, as previously described (Padilla-Benavides et al., 2013a; Padilla-Benavides and Arguello, 2016). The unbound Cu^+^ was removed by washing the samples in 10 kDa cut-off centricons. Protein concentrations were determined using Bradford assay prior to mineralization of the samples using 35% HNO_3_ (trace metal grade) for 1 h at 80°C followed by neutralization using 3% H_2_O_2_. Cu bound to the protein was measured by atomic absorption spectrometry (AAS). Stoichiometry of Cu^+^ bound to the PiC2A and PiC2B proteins was calculated by normalizing Cu^+^ content to protein concentration and then normalizing holo-protein Cu/protein ratios and subtracting the background of the apo-protein Cu/protein ratios.

Ultra-trace Cu analysis method - The method for Cu ultra-trace (< 1 ppm) analysis of all samples was adapted from a previously described protocol (Tavera-Montanez et al., 2019). Briefly, Cu quantification was carried out using an atomic absorption spectrometer PerkinElmer AAnalyst 800 with a Cu hollow cathode lamp as the radiation source. The AAS was equipped with a graphite furnace (GF-AAS) and UltraClean THGA^®^ graphite tubes (PerkinElmer). This technique allows for accurate ultra-trace copper analysis with limited volume samples, minimizing dilution of samples. For accurate and contaminant-free measurements, all analytical glassware and consumables were acid washed overnight in 5% (v/v) hydrochloric acid and rinsed with 18 MΩ purified water before use. Cu standard solutions were prepared from a 1,000 mg/L solution (Sigma-Aldrich) to obtain a calibration curve and determine the dynamic range of the method. The limit of detection for Cu, computed as three times the standard deviation of the intercept of the calibration (3σ), was 10 ppb, and the limit of linearity (LOL) was established at 300 ppb. In a typical analysis of a sample, a known mass of sample was digested in concentrated nitric acid using single-stage digestion. The resulting solution was analyzed for copper in the AAS with measurements carried out at least in triplicates. Cu content on each sample was normalized to the initial mass of protein per sample.

Cu^+^ transfer experiments – Cu^+^ transfer from the His-tagged metallated holo-PiC2A to apoCuA was performed similarly to previous experiments using Cu^+^-ATPases, with minor modifications (Padilla-Benavides et al., 2013a; Blaby-Haas et al., 2014; Padilla-Benavides et al., 2014; Padilla-Benavides and Arguello, 2016). Briefly, PiC2A was loaded or not with Cu^+^, as described above, and bound to a Ni^2+^-nitrilotriacetic acid column in buffer. Apo-CuA was used as a Cu^+^ acceptor. Proteins were allowed to interact for 15 min at RT and proteins were then separated by washing with 25 mM Hepes pH 8.0, 100 mM sucrose, 500 mM NaCl, 0.01% DDM, 0.01% azolectin, 10 mM ascorbic acid, and 20 mM imidazole followed by elution with 25 mM Hepes pH 8.0, 100 mM sucrose, 500 mM NaCl, 0.01% DDM, 0.01% azolectin, 10 mM ascorbic acid, and 300 mM imidazole. Cu^+^ content was determined by the Ultra-trace copper analysis method as described above. Controls were performed where apo-PiC2 was incubated with apoCuA and then subjected to the same procedure.

Immunoprecipitation - Proliferating and differentiating primary myoblasts grown in the presence or absence of CuSO_4_ were washed 3 times with ice-cold PBS and resuspended in freshly made IP lysis buffer (50 mM Tris-HCl, pH7.5, 150 mM NaCl, 1% Nonidet P-40, 0.5% sodium deoxycholate, and Complete protease inhibitor). Cell extracts were incubated with the anti-PiC2 and anti-COX1 primary antibodies at 4°C for 2 h, followed by an overnight incubation with Pure-Proteome Protein A/G mix magnetic beads (Millipore Sigma). Samples were washed as indicated by the manufacturer, and immunoprecipitated proteins were eluted in freshly prepared IP-elution buffer (10% glycerol, 50 mM Tris-HCl, pH 6.8, and 1 M NaCl) at RT for 1 h, as previously described (Padilla-Benavides et al., 2010). Samples were analyzed by western blot probing for the antibodies indicated in the figures.

Metalloproteomic analyses - Analysis of cuproproteins of differentiating murine myoblasts was performed by 2-dimension grazing-exit x-ray fluorescence (2D-GE/XRF) coupled to liquid chromatography-mass spectrometry (LC-MS/MS) as previously described (Raimunda et al., 2012; Raimunda et al., 2013). Briefly, 100 µg of whole cell extract of primary myoblasts differentiated for 24 h in the presence or absence of insulin and Cu were resolved in 10% native polyacrylamide gels. Denaturing agents were not used for sample preparation or electrophoresis. Gels were blotted onto PVDF membranes using a wet transfer system and blots were analyzed using synchrotron micro-XRF at the GSECARS beamline 13-ID-E, at the Advanced Photon Source (APS), Argonne National Lab (Illinois, United States). The incident X-ray beam energy was tuned to 10.2 keV for these analyses, and focused to a relatively broad spot size of ∼150 × 200 mm using rhodium-coated silicon mirrors in a Kirkpatrick-Baez geometry (Sutton et al., 2017). Energy dispersive X-ray fluorescence spectra was collected using a four-element silicon drift detector (Vortex ME4, SII NanoTechnology). Spectra were collected in mapping mode (20 mm × 70 mm maps) by raster scanning the beam through the incident beam in a continuous scan mode so that maps have a 250 mm pixel size with an accumulation time of 80 ms per pixel. Maps of total measured Cu Ka fluorescence intensity were generated and normalized to incident flux, using the GSECARS Larch software (Newville, 2013). PVDF membranes were marked at the edges of the blot and the images obtained using micro-XRF mapping were superimposed onto the membranes. The bands corresponding to the Cu signal were excised for tryptic digestion and identification by LC-MS/MS at the University of Massachusetts Chan Medical School, Proteomics and Mass Spectrometry Facility. Data was analyzed with the software Scaffold_3.5.1 (Proteome Software Inc.).

Statistical analysis - Statistical analyses were performed using Kaleidagraph (Version 4.1). Data comparisons and statistical significance were determined using one-way analysis of variance (ANOVA). Experiments where *p <* 0.05 were considered statistically significant.

## 3 Results

### 3.1 Expression of *mPiC2* is induced during primary myoblast differentiation

**FIGURE 1:**
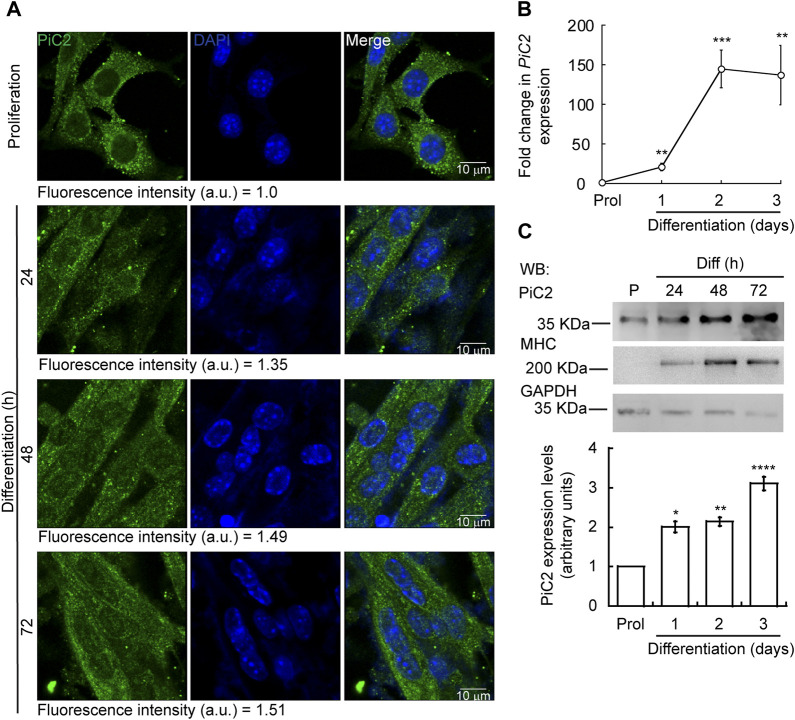
Expression of mPiC2 increases over the course of differentiation in primary myoblasts derived from mouse satellite cells. **(A)** Representative confocal microscopy analysis of PiC2 expression and localization. Proliferating (48 h) and differentiating (24 h, 48 h, and 72 h) primary myoblasts were fixed, and stained with a fluorescent antibody against PiC2 (green) and DAPI (blue). **(B)** *mPiC2* mRNA expression is induced during differentiation. qRT-PCR analysis of *mPiC2* mRNA levels. Values normalized to *Ef1α* and non-differentiated (Prol) controls, *n* = 3. **(C)** mPiC2 protein expression is induced during differentiation. Representative western blot of PiC2 protein levels (top panel) and quantification (bottom panel). Membranes were blotted with PiC2, the differentiation marker myosin heavy chain (MHC), and GAPDH was used as loading control. *n* = 3 All values are reported as means ± SEM. Significance was determined by two-way ANOVA; *p < 0.05, **p < 0.01, ***p < 0.001 and ****p < 0.0001 compared to Prol cells.

Differentiating myoblasts have a high demand for Cu and, as such, require an increase in the levels of imported Cu and mobilized within the cell (Vest et al., 2018; Tavera-Montanez et al., 2019). To assess whether mPiC2 may play a role in Cu transport during myogenesis, its expression and distribution in differentiating primary myoblasts was assessed (Figure 1). Confocal microscopy analysis revealed a punctate cytosolic staining pattern for mPiC2, that was preserved from proliferating myoblasts to differentiated myotubes (Figure 1A). *mPiC2* mRNA levels increased 25-fold in day 1 differentiated myoblasts compared to proliferating controls (Figure 1B). *mPiC2* expression increased further to approximately 150-fold higher than controls at days 2 and 3. Western blot analyses show mPiC2 expression was also upregulated in response to the induction of differentiation, with the highest protein levels identified 72 h after inducing differentiation **(** Figures 1C,D). These data show that PiC2 expression is induced during differentiation, suggesting that it may transport Cu during myogenesis.

### 3.2 *mPiC2* expression is enhanced by the addition of Cu and is controlled by MTF1 binding in differentiating primary myoblasts

**FIGURE 2:**
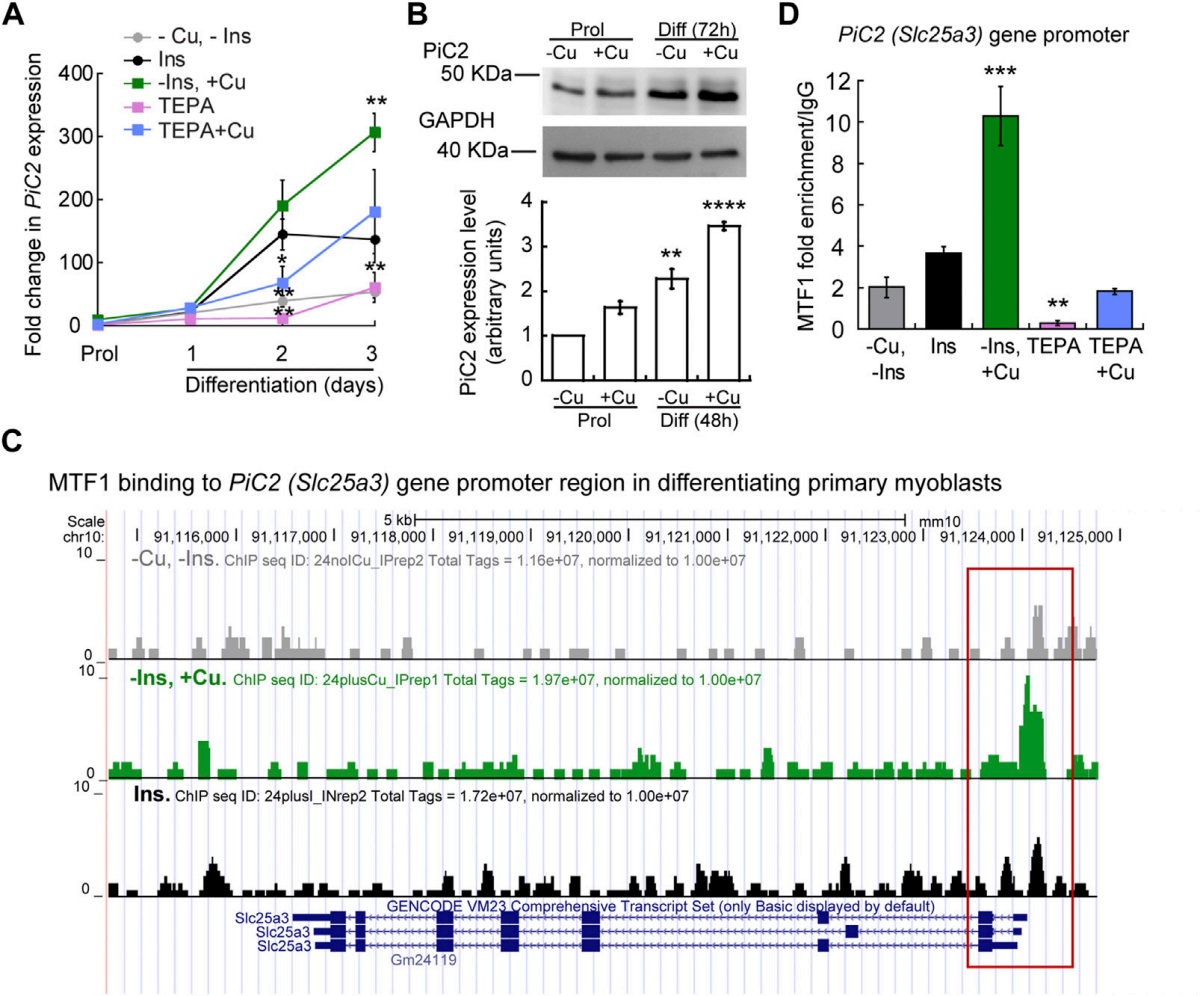
*mPic2* is a target gene of MTF1 in differentiating primary myoblasts, and Cu promotes this binding. **(A)** mRNA levels of *mPiC2* in proliferating and differentiating primary myoblasts. *mPic2* expression was measured by qPCR. Values normalized to *Ef1α* and non-differentiated (Prol) controls, *n* = 3. **(B)** Representative western blot (top) and quantification (bottom) of proliferating (48 h) and differentiating (72 h) primary myoblasts grown in the presence or absence of Cu. Values normalized to GAPDH and non-treated Prol controls, *n* = 3. **(C)** Genome browser tracks of ChIP-seq experiments assessing MTF1 binding to the *mPic2* promoter. Promoter region of *mPic2* shown in red box. **(D)** ChIP-qPCR validation of MTF1 binding the *mPic2* promoter region. MTF1 enrichment at the *mPic2* promoter was assessed by ChIP-qPCR, *n* = 3. All values are reported as means ± SEM. Significance was determined by two-way ANOVA; **p < 0.01 and ***p < 0.001 compared to cells differentiated with insulin. Abbreviations used are: cells differentiated in the absence of insulin and copper (-Ins, −Cu), with insulin (Ins), of insulin and 30 µM CuSO_4_ (-Ins, +Cu), with the 30 µM of the Cu-chelator TEPA (TEPA), and with 30 µM of TEPA supplemented with 30 µM CuSO_4_ (TEPA + Cu).

To further explore the potential of mPiC2 as a Cu transporter during myogenesis, we next assessed the impact of Cu on *mPiC2* expression. Cultured primary myoblasts can be differentiated *via* serum deprivation and insulin supplementation. In the absence of insulin, the myoblasts differentiate poorly (Conejo et al., 2001). However, differentiation can be restored by the addition of Cu to the growth medium. By contrast, Cu chelation by TEPA impaired differentiation even in cells grown in the presence of insulin (Vest et al., 2018). Moreover, Cu has been shown to induce the expression of lineage specific genes, such as *Pax7, myogenin,* and *myosin heavy chain*, thereby promoting the growth and differentiation of cultured primary myoblasts (Vest et al., 2018). To determine whether mPiC2 expression was impacted by cellular Cu levels, primary myoblasts were treated with TEPA and/or non-toxic concentrations of Cu in the presence or absence of insulin in the culture medium (Figure 2). Myoblasts grown in the presence of insulin and the absence of Cu (black) are considered the normal differentiation control. *PiC2* expression increases upon differentiation, with mRNA levels at day 3 roughly 150-fold higher than those observed in proliferating (Prol) cells (Figure 2A). Cells grown in media depleted of insulin (gray), by contrast, show a modest rise in *mPiC2* expression level compared to Prol control cells. Notably, Cu treatment in the absence of insulin (green) is sufficient to induce *mPiC2* expression to a level even higher than that of the normal differentiation control: roughly 300-fold higher than the Prol cells at day 3 of differentiation compared to 150-fold higher, respectively. Conversely, treatment with TEPA (pink) decreased *mPiC2* expression compared to the differentiation control, with the levels at day 3 of differentiation on par with that of cells grown in the absence of both insulin and Cu. Cu treatment following TEPA treatment (blue) rescues the phenotype, with *mPiC2* expression levels approximately the same as those observed in the differentiation control. A representative western blot of PiC2 expression and quantification for proliferating and differentiating myoblasts showed a similar trend of an increased expression of PiC2 upon Cu supplementation (Figure 2B).

In conjunction with Cu, the metal-sensing transcription factor MTF1 has been shown to regulate gene expression during myogenesis (Tavera-Montanez et al., 2019). Several myogenic genes (i.e., *myogenin*, *MyoD*) are known targets of MTF1, which binds their promoter regions during differentiation to activate their expression. The addition of Cu enhances this effect (Tavera-Montanez et al., 2019). To determine whether MTF1 binds *mPiC2* during differentiation, previously generated chromatin immunoprecipitation sequencing (ChIP-seq) data for MTF1 binding (Tavera-Montanez et al., 2019) was accessed and read enrichment over the *PiC2* gene was examined (Figure 2C). MTF1 was enriched at the *PiC2* promoter region (indicated in red) in cells grown in both the presence and absence of insulin, though the addition of insulin enhanced this effect. MTF1 enrichment was most striking in cells differentiated in the presence of Cu. These results were validated by ChIP-qPCR (Figure 2D). MTF1 enrichment at the *PiC2* promoter was approximately 2-fold higher in cells grown in the presence of insulin compared to those grown in the absence of it. The addition of Cu to medium lacking insulin enhanced MTF1 enrichment 8-fold compared to cells grown in the absence of insulin and Cu and 6-fold compared to cells grown in the presence of insulin and the absence of Cu. Interestingly, the promotor of *mPiC2* contains three potential metal responsive elements (MRE) for MTF1 characterized by the 5′-TGCRCNC-3′ consensus sequence (Heuchel et al., 1994; Langmade et al., 2000; Miles et al., 2000; Andrews et al., 2001; Wang et al., 2004; Selvaraj et al., 2005). The primers used here were designed to cover two of these MREs. To further show that Cu specifically influenced MTF1 binding to the *mPiC2* promoter region, cells grown in the presence of TEPA were analyzed. TEPA treatment decreased MTF1 enrichment markedly, to a level even lower than that observed in cells grown in the absence of both insulin and copper. Cu treatment following TEPA treatment returned MTF1 enrichment levels to those observed in cells grown in the absence of both insulin and Cu. Taken together, these data show that *mPiC2* is a target of MTF1 during primary myoblast differentiation, and that addition of Cu to the growth medium enhances MTF1 enrichment at the *mPiC2* promoter.

### 3.3 Recombinant hPiC2 binds Cu^+^ *in vitro*

**FIGURE 3:**
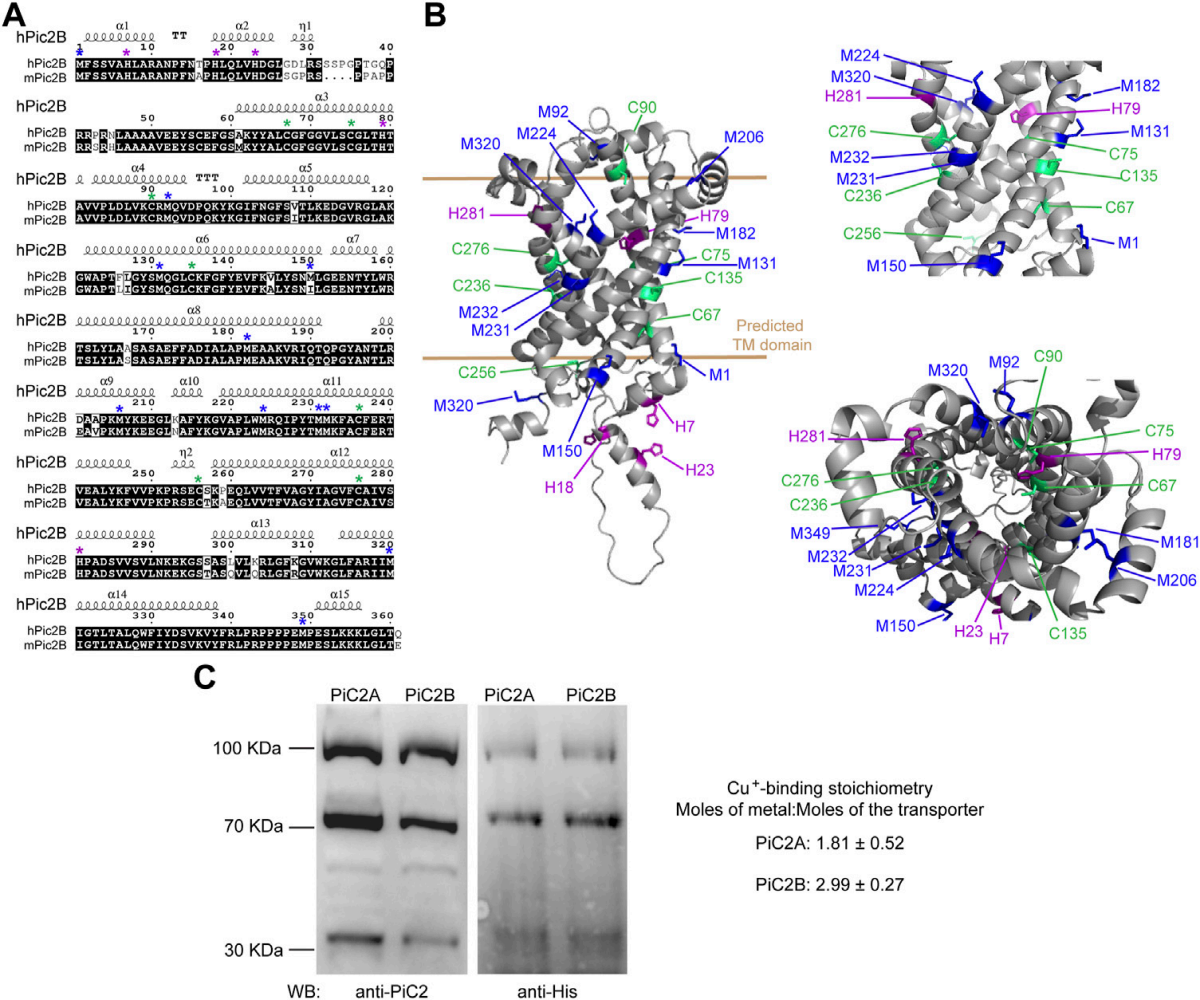
mPiC2 protein has several potential Cu binding sites. **(A)** Sequence homology comparison of the human hPiC2B and murine mPiC2B. Alignment of hPiC2B and mPiC2B amino acid sequences. Areas of homology between the sequences of the two transporters are indicated in black, potential Cu^+^-binding resides indicated with green asterisks for cysteines, blue asterisks for methionines and purple asterisks for histidines (*). **(B)** Representative model of mPic2 protein and potential Cu^+^-binding sites obtained from Alpha fold. Potential Cu^+^-binding residues indicated in green. **(C)** Representative western blots of recombinant PiC2A and PiC2B protein expression detected by both anti-PiC2 antibody and anti-His tag antibody. Monomeric, dimeric, and trimeric forms are detected by both antibodies. Cu^+^-binding stoichiometry of Pic2A and Pic2B. *n* = 3, results reported as means ± SEM.

Sequence analysis of yeast yPiC2 has shown that it is partially homologous to mammalian SLC25A3. The mammalian SLC25A3, or PiC2, has two isoforms: PiC2A and PiC2B. Analysis of the amino acid sequences of mPiC2B and the human hPiC2B show that they share a high level of homology (Figure 3A; residues highlighted in black). Previous sequence analyses and prediction of the TM segments the bovine SLC25A3, bPiC2, suggested the protein has six TM domains (Runswick et al., 1987) and a significant number of conserved Cys, Met, and His residues within these domains, which could indicate possible Cu binding sites. These residues (indicated in Figure 3A with green, blue and purple asterisks, respectively; and indicated in Figure 3B in the same colors) are also highly conserved in both the human hPiC2B and murine mPiC2B. Figure 3B shows the putative structure of hPiC2B modeled using AlphaFold. Several putative ligands could provide transient binding sites for transporting the copper ions. Cu binding experiments using the two purified human PiC2 isoforms (Figure 3C) revealed that hPiC2A, had a binding stoichiometry of 2 Cu^+^ per protein, while the other isoform, hPiC2B, had a stoichiometry of approximately 3 Cu^+^ per protein. These findings are in agreement with the AlphaFold prediction that reveals different arrangements that could provide 2–3 Cu^+^-binding sites. Preliminary sequence analysis suggested that PiC2 can be found as a monomer, dimer and trimer, therefore, the orientation of the TM residues may play a role in the formation of these multimeric complexes, and may represent additional binding sites for Cu.

### 3.4 PiC2 interacts with COX and other mitochondrial cuproproteins in primary myoblasts

**FIGURE 4:**
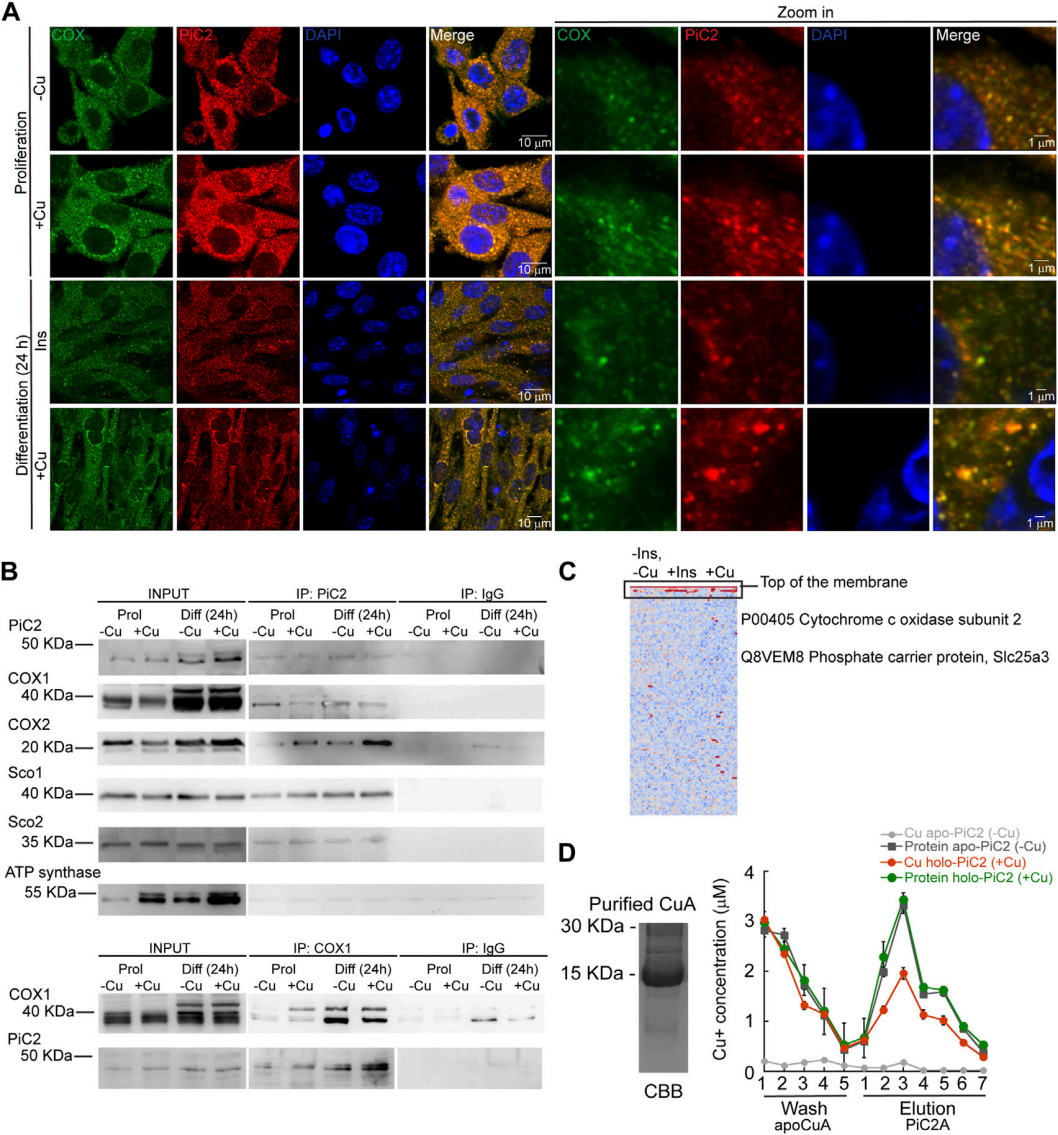
mPiC2 interacts with cytochrome c oxidase and other mitochondrial cuproproteins in primary myoblasts derived from mouse satellite cells. **(A)** mPiC2 co-localizes with COX. Confocal imaging showing mPiC2 (red) and COX (green) co-localization (yellow). The nucleus is stained with DAPI (blue). Set of four panels on the right are zooms of areas in the set of four panels on the left. **(B)** Co-IP of mPiC2 with mitochondrial cuproproteins. Proliferating and differentiating primary myoblasts were treated with or without Cu. Co-IP was performed with and anti-PiC2 antibody and probed against PiC2 and COX1, COX2, Sco1, and Sco2; the ATP synthase was used as a negative control. Reciprocal co-IP using an anti-COX1 antibody was probed with an anti-COX1 and anti-PiC2 antibodies. **(C)** Synchrotron based X-Ray fluorescence analyses couple to protein sequencing by mass spectrometry of native PAGE gels from whole cell extracts of differentiating primary myoblasts showed the presence of mPiC2 and COX in a high molecular band that contained copper (indicated by black box). **(D)** Cu^+^ transfer from metallated PiC2A to apo-CuA. Cu^+^ concentration is shown in red, and the corresponding protein is shown in green. Control Cu^+^ transfer experiment is shown in pale (Cu^+^ signal) and dark gray (protein signal). In all cases, the contents of the wash and elution fractions are shown. The data presented in this figure corresponds to three independent biological replicates shown as the mean for Cu^+^ and protein concentration ±SEM.

COX requires Cu for proper activity and function. Inactivation of PiC2 in both yeast and mice results in disruptions of the COX activity and decreases in mitochondrial Cu content (Vest et al., 2013; Boulet et al., 2018; Zhu et al., 2021). This suggests that PiC2 may interact with COX or other mitochondrial Cu^+^-binding proteins, such as Sco1 and Sco2, to support the metallation of the oxidase. To test this, the localization of mPiC2 relative to the mitochondrial COX in differentiating primary myoblasts was analyzed. Consistent with the data shown in Figure 1A, mPiC2 staining (red) showed a punctate cytosolic pattern during both proliferation and differentiation (Figure 4A). Moreover, the staining pattern of mPiC2 was indicative of a mitochondrial distribution of the protein, which was further confirmed by its co-localization with COX (green staining; co-localization indicated by yellow staining). Strikingly, the co-localization of mPiC2 with COX appears to be enhanced by the addition of Cu to the growth medium. Pearson correlation coefficient for the regions of interest zoomed-in in Figure 4 showed a positive association between PiC2 and the oxidase. In the case of proliferating cells grown in the absence of Cu *r* = 0.88, and for those grown with Cu *r* = 0.91. In the case of differentiating cells, the Pearson correlation was *r* = 94 and *r* = 90 for cells differentiated in the absence and presence of Cu, respectively. To further investigate the potential interaction between PiC2 and COX, we performed co-immunoprecipitation (co-IP) experiments in proliferating and differentiating myoblasts in the presence and absence of Cu (Figure 4B). As expected, more mPiC2 was pulled down in the differentiated samples, consistent with an increase in the expression of mPiC2 upon differentiation. Pull down of mPiC2 followed by probing for two of the COX subunits, COX1 and COX2, revealed that both subunits are present on the gel, indicating an interaction with mPiC2. Moreover, probing for additional mitochondrial cuproproteins Sco1 and Sco2 also resulted in protein signal, showing that these proteins also associate with mPiC2 (Figure 4B). Reciprocal immunoprecipitation experiments with proliferating and differentiating myoblasts cultured on similar conditions, but where Cox1 was pulled down, showed that the interaction with mPiC2 is maintained (Figure 4B). These data suggest that mPiC2 interacts with at least two types of mitochondrial proteins involved in Cu transport and homeostasis. We confirmed the interaction between Cu^+^, PiC2, and COX using x-ray fluorescence-mass spectrometry (XRF/MS; Figure 4C). Whole cell extracts of differentiating myoblasts treated with or without insulin or Cu were separated by non-denaturing PAGE to preserve protein-Cu interaction prior to XRF-MS. Samples were analyzed for the presence of Cu, and a high molecular weight band was detected (Figure 4C, indicated by black box). Mass spectrometry sequence analyses showed that COX was the only known cuproprotein identified in the samples, however, PiC2 was also recognized. *In vitro* Cu^+^ transfer experiments using apo-CuA and PiC2A showed that the metallated transporter is capable of donating Cu^+^ to the oxidase (Figure 4D), confirming that this transfer is thermodynamically favored. Together, these results demonstrate a previously unknown interaction between PiC2 and mitochondrial cuproproteins, further supporting a role for PiC2 in Cu transport during myogenesis.

### 3.5 mPiC2 is required for the growth and differentiation of primary myoblasts derived from mouse satellite cells

**FIGURE 5:**
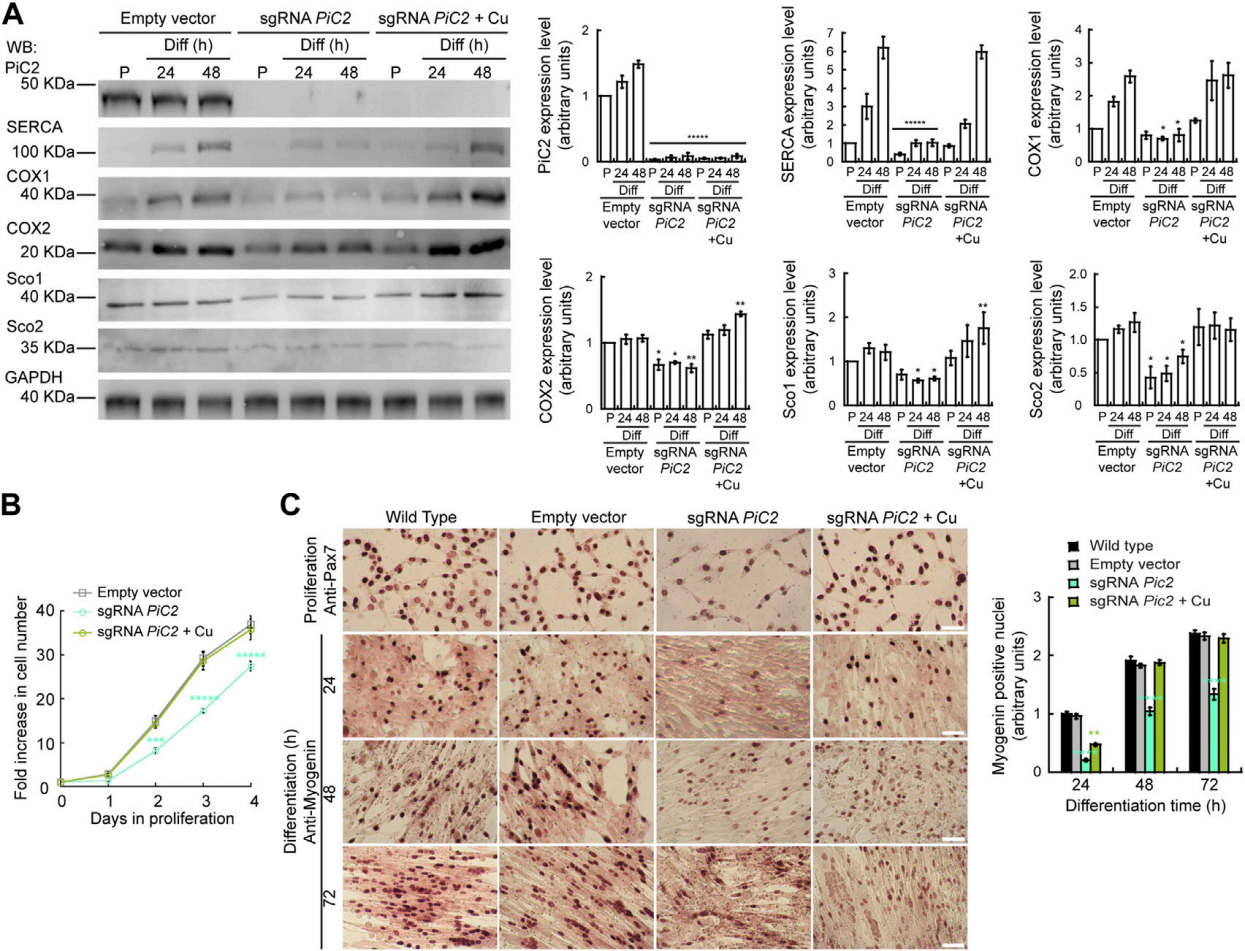
Deletion of *mPiC2* impairs the proliferation and differentiation of primary myoblasts derived from mouse satellite cells. **(A)** Deletion of mPiC2 reduces the expression of mitochondrial cuproproteins. Representative western blot of mPiC2 and mitochondrial cuproproteins in control cells (empty vector) or cells in which CRISPR/Cas9 was used to delete mPiC2 (sgRNA *PiC2*) or KO cells supplemented with CuSO_4_ (sgRNA *PiC2* + Cu) during proliferation (P) and at 24 h and 48 h after inducing differentiation. GAPDH was used as a loading control. Plots represent the quantification of each protein analyzed from three independent biological replicates. Values are the means ± SEM. Significance was determined by two-way ANOVA; *p < 0.05, **p < 0.01 and ****p < 0.0001 *vs.* empty vector at the corresponding timepoint. **(B)** Deletion of mPiC2 delays proliferation. Cell counting assay of empty vector, sgRNA against *PiC2*, and sgRNA *PiC2* + Cu cells at different points during proliferation. *n* = 3. All values are reported as means ± SEM. Significance was determined by two-way ANOVA; ***p < 0.001 and ****p < 0.0001 compared to empty vector cells. **(C)** Deletion of mPiC2 delays myogenesis. Staining of wild type, empty vector, sgRNA *mPiC2*, and sgRNA *mPiC2* + Cu cells with either anti-Pax7 antibody (marker of proliferation) or anti-myogenin antibody (marker of differentiation). Associated plot represents the number of myogenin positive nuclei of cells undergoing differentiation at the indicated timepoints in the immunohistochemistry images *n* = 3. All values are reported as means ± SEM. Significance was determined by two-way ANOVA; ***p < 0.001 and ****p < 0.0001 compared to empty vector cells.

Dysfunction and loss of activity of mPiC2, in addition to leading to defects in COX activity, has also been linked to rare diseases, such as fatal and benign infantile myopathies, lactic acidosis, hypertrophic cardiomyopathy, and muscular hypotonia (Mayr et al., 2011; Boulet et al., 2018). Mutations in mPiC2 have been shown to lead to disruptions in P_i_ transport that impacts muscle function and development (Mayr et al., 2007; Mayr et al., 2011). To assess the link between mPiC2 function and myogenesis, we used CRISPR/Cas9 to knock out (KO) *mPiC2* in primary myoblasts derived from mouse satellite cells. Western blotting shows that *mPiC2* KO was successful, with effectively no mPiC2 protein signal in the KO cells in both the presence and absence of Cu compared to the control cells transduced with the empty vector (Figure 5A). Notably, *mPiC2* KO had a large impact on the abundance of the mitochondrial cuproproteins, COX1, COX2, Sco1, and Sco2 (Figure 5A). Addition of Cu to the culture media reverted the impact of this deletion, restoring the protein levels. This effect on COX expression was more pronounced in differentiated cells than in proliferating cells.

Thus far, we have found that mPiC2 expression is upregulated upon differentiation in primary myoblasts and that this effect is enhanced by the addition of Cu to the culture medium (Figure 1, Figure 2A). Analysis of the effect that *PiC2* KO has on primary myoblasts revealed that deletion of the transporter also impacts proliferation, and these cells present with a growth defect compared to control cells (Figure 5B). This delay was reversed by the addition of Cu to the growth medium, with the number of cells actively proliferating now similar to control levels. Similarly, immunostaining with the proliferation marker Pax7 showed fewer myoblasts in a proliferative state upon *mPiC2* deletion compared to controls, with the presence of Cu mitigating this effect (Figure 5C). To determine how *mPiC2* KO affects myogenesis, cells were stained with an anti-myogenin hybridoma, a marker of differentiation (Figure 5A). As with proliferation, *mPiC2* KO resulted in a delay in the progress of differentiation compared to the control cells, an effect that the addition of Cu to the growth medium abolished. This phenotype was also evidenced in western blot analyses where another differentiation marker, the sarcoplasmic reticulum Ca^2+^-ATPase (SERCA; Figure 5A), also presented a decreased expression upon PiC2 deletion. SERCA expression was also recovered upon Cu supplementation. Taken together, these experiments confirm that mPiC2 is a mitochondrial Cu transporter that interacts with cuproproteins to favor maturation of COX. This process favors the development and differentiation of cultured skeletal muscle cells.

## 4 Discussion

Mitochondrial function and, subsequently, cellular energy production are reliant in part on Cu. While Cu transport within the mitochondria has been characterized, the delivery of Cu to the mitochondria through the Cu-impermeable mitochondrial membranes is incompletely understood. Studies in yeast identified a mitochondrial phosphate transporter, yPiC2, that is necessary for the import of Cu into the mitochondria and the metallation of COX (Vest et al., 2013). The closest mammalian homolog of this transporter, SLC25A3 (PiC2), has mainly been characterized as a mitochondrial transporter of P_i_. Our studies in murine primary myoblasts derived from mouse satellite cells, an ideal system for studying mitochondrial biogenesis and transport, have uncovered a role for PiC2 in this system.

Muscle tissue has an abundance of mitochondria, as these tissues have high energy requirements. This subsequently leads to a high demand in Cu to metalate cuproproteins involved in energy production and mitochondrial function, such as COX (Vest et al., 2013). To meet these needs, myoblasts must mobilize additional Cu to the mitochondria. We found that mPiC2 expression is induced in primary myoblasts upon differentiation. This finding suggests a role for PiC2 in myogenesis, though whether this role was related to Cu delivery or not was yet unclear.

In addition to being required for the induction of myogenesis, Cu is also implicated in the transcriptional regulation of myogenic genes (Tavera-Montanez et al., 2019). Myoblast differentiation is induced *in vitro* by serum starvation and supplementation of insulin into the culture medium, and the lack of insulin in the medium results in cells that differentiate poorly. We show that while insulin depletion impedes the upregulation of *mPiC2* in differentiating myoblasts, addition of Cu to the growth medium restores the expression of the transporter to that observed in cells supplemented in insulin. Conversely, depletion of Cu *via* the chelator TEPA resulted in *mPiC2* expression levels on par with those observed in insulin-deprived myoblasts, indicating cells without Cu differentiate poorly. Adding Cu back following chelation with TEPA reverses this effect. These data together indicate that Cu is both necessary for the differentiation-induced increase in *mPiC2* expression and sufficient to ameliorate the effects of insulin-deprivation on the differentiated myoblasts.

We have previously described the metal-sensing transcription factor MTF1 as playing a key role in myoblast differentiation. MTF1 binds the promoter regions of myogenic genes to induce their expression, and this event is enhanced by the addition of Cu to the myoblast growth medium (Tavera-Montanez et al., 2019). Similarly, we observed a marked enrichment in MTF1 binding to the promoter region of the *mPiC2* gene during myogenesis in the presence of Cu. This enrichment is even several-fold higher than that observed in differentiating myoblasts induced by the addition of insulin, suggesting the presence of Cu additionally enhances MTF1 binding to the *mPiC2* promoter. Depletion of Cu greatly impedes the binding of MTF1 to this promoter, and supplementation of Cu to the medium following Cu depletion restores partially the binding to roughly 50% of that seen in insulin-induced cells. These data suggest that enhanced MTF1 binding to the *mPiC2* promoter during differentiation could cause the RNA- and protein-level increases in mPiC2 expression observed in differentiated cells and that binding of MTF1 to the promoter region is promoted by the addition of Cu. In this way, the overall cellular increase in Cu can facilitate the delivery of Cu to specific compartments, such as the mitochondria, to be loaded into proteins that require Cu as a cofactor for their enzymatic activity.

For PiC2 to act as a Cu^+^-transporter, the protein needs to contain residues that coordinate Cu binding, such as Cys, His, and Met. yPiC2, the first SLC25A3 to be identified as a Cu transporter, has several such residues, many of which are conserved in the mammalian homologs. Sequence analysis between the human and mouse B isoforms of PiC2 (hPiC2B and mPiC2B, respectively) revealed conserved residues and domains that are common to other Cu transporters. Homology modeling of mPiC2B revealed three potential metal binding sites, which were validated by biochemical analyses showed that PiC2 is, indeed, a Cu^+^-binding protein. Despite PiC2 has been shown to translocate Cu from the IMS to the matrix, it has been speculated that it could also play a role in transporting Cu along both directions (Cobine et al., 2021).

The current understanding of COX metallation involves the soluble protein, Cox17, and two proteins located in the inner membrane of the mitochondria, Sco1 and Sco2 (Buchwald et al., 1991; Horng et al., 2005; Banci et al., 2008a; Banci et al., 2008b; Leary et al., 2009). As of yet, there has been no mechanism describing how Cu gets in through the outer mitochondrial membrane to these proteins in order to metalate COX. Based on the fundamental principle that there is no labile Cu in cells due to its reactivity, it is therefore necessary that Cu is transported from donor to acceptor proteins *via* direct interaction. Cu^+^-transfer to the final destination within the cell is driven by affinity gradients (Banci et al., 2010). The experiment *in vitro* showing that hPic2 can transfer Cu^+^ to COX2 suggests that the transporter could be involved upstream in the copper transfer chain at the IMS. Moreover, since it has been shown that copper supplementation can rescue a COX2 deficient phenotype induced by mutations in Sco2 (Salviati et al., 2002), it is important to show that (despite less efficiently) copper delivery can occur even when the essential Sco2 protein is not functional. Whether or not mitochondrial Cu^+^ delivery *via* PiC2 is in any way coupled to mitochondrial P_i_ transport is unknown and requires additional study.

mPiC2 expression was significantly upregulated during myoblast differentiation, which suggests that the transporter is required during myogenesis but does not directly prove this function. CRISPR/Cas9-mediated deletion of *mPiC2* in myoblasts resulted in the reduction in the expression of COX1, COX2, Sco1, and Sco2, as well as the delayed entry of the cells into both proliferation and differentiation states. These data indicates that mPiC2 expression contributes to normal myoblast growth and differentiation. Interestingly, the supplementation of Cu to the culture medium of *mPiC2* KO myoblasts completely reversed these effects. Despite the fact that mPiC2 seems to contribute to this process, PiC2 is not an essential protein during muscle maturation and therefore, additional mechanisms, such as COX17, must be in place. This finding strongly suggests that the driving force behind the deleterious effects of *mPiC2* KO is the lack of Cu delivery to mitochondria. However, this does imply that Cu may still be getting into the impermeable outer mitochondrial membrane even in the absence of mPiC2. This phenomenon will require further investigation.

Taken together, these data demonstrate that mPiC2 expression is required both for the proper growth of proliferating myoblasts and the progression of myogenesis. *mPiC2* expression is upregulated during the differentiation of primary myoblasts, likely enhanced by the binding of MTF1 to the promoter region of the *mPiC2* gene. A possible role for mPiC2 in the copper transport chain that leads to the metalation of these proteins. Deletion of *PiC2* results in the downregulation of mitochondrial cuproproteins, such as COX, as well as the delay of both proliferation and differentiation. Consistent with previous studies (Boulet et al., 2018), the addition of Cu to the growth medium is sufficient to offset the effects of *mPiC2* deletion, suggesting that the transporter plays a role in the network of Cu homeostasis in myoblasts. This study provides a foundation for additional study of mitochondrial Cu delivery and the role that Cu plays in both mitochondrial biogenesis and myogenesis in muscle tissue.

## Data Availability

The datasets analyzed for this study can be found in the GEO database with accession number: GSE116331, and can be downloaded here: https://www.ncbi.nlm.nih.gov/geo/query/acc.cgi?acc=GSE116331.
